# Chronic pain diagnosis in refugee torture survivors: A prospective, blinded diagnostic accuracy study

**DOI:** 10.1371/journal.pmed.1003108

**Published:** 2020-06-05

**Authors:** Gunisha Kaur, Roniel Weinberg, Andrew Robert Milewski, Samantha Huynh, Elizabeth Mauer, Hugh Carroll Hemmings, Kane Owen Pryor

**Affiliations:** 1 Department of Anesthesiology, Weill Cornell Medicine, New York, New York, United States of America; 2 Department of Healthcare Policy and Research, Weill Cornell Medicine, New York, New York, United States of America; Johns Hopkins University Bloomberg School of Public Health, UNITED STATES

## Abstract

**Background:**

An estimated 87% of torture survivors experience chronic pain such as brachial plexopathy from upper extremity suspension or lumbosacral plexus injury from leg hyperextension. However, a vast majority of pain is undetected by evaluators due to a lack of diagnostic tools and confounding psychiatric illness. This diagnostic gap results in exclusive psychological treatment rather than multimodal therapies, substantially limiting rehabilitation. We hypothesized that the United Nations Istanbul Protocol (UNIP) would have a sensitivity of approximately 15% for pain detection, and that the use of a validated pain screen would improve its sensitivity by at least 29%, as compared to the reference standard (pain specialist evaluation).

**Methods and findings:**

This prospective blind-comparison-to-gold-standard study of survivors of torture, as defined by the World Medical Association, took place at Weill Cornell Medicine between February 1, 2017, and June 21, 2019. 11 women and 9 men, for a total of 20 participants, were included in the analysis. Five participants received 2 UNIP evaluations, for a total of 25 unique evaluations included in the analysis. Participants were representative of a global population, with home countries in Africa, Central America, South Asia, the Caribbean, and the Middle East. Methods of torture experienced were homogeneous, following the predictable pattern of systematic torture. Participants first received the standard evaluation protocol for torture survivors (UNIP) by a trained evaluator, and subsequently received a validated pain screen (Brief Pain Inventory–Short Form [BPISF]) followed by a noninvasive examination by a pain specialist physician (reference standard). The primary outcome was the diagnostic and treatment capability of the standard protocol (index test) versus the validated pain screen (BPISF), as compared to the reference standard. Trained evaluators performing the initial assessment with the UNIP (index test) were blinded to the study, and the pain specialist physician (reference standard) was blinded to the outcome of the initial UNIP evaluation and the BPISF; data from the initial UNIP assessment were not gathered by the principal investigator until all other study procedures were completed. Providers using only the UNIP captured pain in a maximum of 16% of evaluations, as compared to 85% of participants being diagnosed with pain by the reference standard. When employed, the validated pain screen had a sensitivity of 100% (95% CI 72%–100%) and a negative predictive value of 100%, as compared to a sensitivity of 24% (95% CI 8%–50%) and a negative predictive value of 19% (95% CI 5%–46%) for the index test. The difference in the sensitivity of the UNIP as compared to the BPISF was significant, with *p* < 0.001. No adverse events owing to participation in the study were reported by participants. Limitations of the study include small sample size, its single-site nature, and the exclusion of individuals who did not speak 1 of the 5 study languages.

**Conclusions:**

These data indicate that a validated pain screen can supplement the current global standard assessment of torture survivors, the UNIP, to increase the accuracy of pain diagnosis.

**Trial registration:**

ClinicalTrials.gov NCT03018782.

## Abstract 2

Gunisha Kaur and co-workers investigate diagnosis of chronic pain in people who have survived torture.

## Author summary

**Why was this study done?:**

- The number of refugee torture survivors worldwide is increasing as violent global conflict accelerates.
- Refugee torture survivors often experience chronic, debilitating pain that is underdiagnosed due to complex, confounding psychiatric illness, which impacts treatment and rehabilitation.
- General evaluators do not have diagnostic tools to assist them in the diagnosis of pain in this complex population.

**What did the researchers do and find?:**

- We conducted a prospective blind-comparison-to-gold-standard study of diagnostic accuracy in torture survivors that took place at Weill Cornell Medicine in New York City between February 1, 2017, and June 21, 2019.
- Following a standard medical, psychiatric, and/or gynecological evaluation according to the guidelines of the United Nations Istanbul Protocol (UNIP), study participants received a validated pain screen and noninvasive physical examination by a pain specialist physician.
- We found that providers using only the UNIP diagnosed pain significantly less frequently than was indicated necessary by the reference standard (pain specialist evaluation), and that the validated pain screen accurately identified pain when utilized.

**What do these findings mean?:**

- The global standard assessment of torture survivors, the UNIP, should be supplemented by the use of a validated pain screen to increase the accuracy of chronic pain diagnosis.
- This would potentially shift the standard medical evaluation of 31 million displaced torture survivors globally.
- Accurately diagnosing pain after torture enhances the ability of providers to treat pain and rehabilitate individuals.
- Further studies are warranted to investigate outcomes of treatment of pain after torture.

## Introduction

Accelerating violent global conflict has resulted in 70.8 million displaced people worldwide [1], an estimated 44% of whom have survived torture [2]. With nearly 45,000 newly displaced people every day, physicians in developed countries are increasingly encountering torture survivors in their practice [3]. While countless mechanisms of torture exist, multiple studies demonstrate the uniformity of systematic physical torture: 10 methods may account for up to 98% of physical torture (S1 Table), which most often includes beating or assault with objects, binding or forced postures, burning or cold water hosing, or electrical shock to sensitive areas [4–7]. As a result of these human rights violations, torture survivors experience concurrent physical and psychological trauma that is sustained for decades [8]. Studies demonstrate a high prevalence of chronic pain, as great as 87% (27 million people globally) [9], in torture survivors that may correlate with the mechanism of injury: falanga (beating the soles of the feet; also known as falaka) results in peripheral neuropathy; suspension from upper extremities is associated with brachial plexopathy; and leg hyperextension is correlated with lumbosacral plexus injury [10–12]. Pain from torture not only persists decades after trauma, but can worsen and cause increased disability [13]. Despite the high prevalence of chronic pain, evaluators fail to diagnose pain in torture survivors. It is most often confounded or eclipsed by psychiatric illnesses such as post-traumatic stress disorder (PTSD), major depression (MD), or somatization [14]. The United Nations Istanbul Protocol (UNIP) [15] is a set of internationally recognized [16] guidelines and procedures on the medical evaluation of torture survivors. As the global standard used for the medical assessment of torture survivors, it is often utilized in conjunction with screens for PTSD and MD. While the protocol acknowledges the significance of pain after torture and provides general recommendations for assessing pain, the guidelines are challenging to operationalize and no evidence-based diagnostic tools are recommended. The problematic gap in the diagnosis of pain results in treatment focused exclusively on psychological rather than multimodal therapies, substantially limiting rehabilitation, placing vulnerable individuals at increased risk for opioid abuse, reducing integration into host countries, and increasing healthcare expenditures due to the use of costly emergency care [17–19].

We conducted a prospective blind-comparison-to-gold-standard study on pain after torture (PainT study), to examine the difference in the diagnostic ability of the standard UNIP versus a validated pain screen, as compared to the reference standard, a pain specialist evaluation. We hypothesized that the novel application in torture survivors of a validated pain screen would supplement the UNIP and improve the sensitivity of pain diagnosis by at least 29%, as compared to the reference standard.

## Methods

### Study design

PainT was a prospective blind-comparison-to-gold-standard study approved by the Weill Cornell Medicine Institutional Review Board (protocol number 1608017472) and registered at ClinicalTrials.gov (NCT03018782). The protocol was conducted according to the Declaration of Helsinki. All participants provided written informed consent in their primary language. Interpreters were utilized as appropriate during study procedures. Data were entered into case report forms and a REDCap database.

All authors assume responsibility for the accuracy and completeness of the data and analysis and for the fidelity of the study to the protocol. The results are reported in accordance with the Standards for Reporting Diagnostic Accuracy Studies (STARD) guidelines (S1 STARD Checklist).

### Study population

Eligible individuals were 18 years or older; spoke English, French, Spanish, Arabic, or Punjabi; and had survived torture as defined by the World Medical Association (WMA) Declaration of Tokyo [20]. Participants were recruited consecutively between February 1, 2017, and June 21, 2019, from the Weill Cornell Center for Human Rights (WCCHR), one of the largest academic medical–legal human rights centers in the United States, where a globally representative population is seen [21]. All participants had consented to being contacted for research purposes. Surveys and consents were translated from English into the 4 other study languages, and back translated into English to ensure fidelity.

### Test methods

All 25 participants received a medical evaluation under usual procedures by a trained WCCHR evaluator. Usual procedures include medical, psychiatric, and/or gynecological evaluations that adhere to international guidelines of the UNIP [15], the index test. The diagnostic ability of the UNIP was determined by 2 investigators who analyzed the clinical conclusion and summary of the UNIP evaluations for use of the word “pain” in the context of physical complaints, and for referral to a provider for pain evaluation or treatment. When no clinical conclusion or summary section was delineated (5 occurrences), the core evaluation was analyzed for use of the word “pain.” These were predetermined positivity cutoffs for the index test. Pain captured by the UNIP was defined as a documented clinical diagnosis of any pain or pain syndrome and referral for further evaluation or treatment. To conservatively maximize the sensitivity of the UNIP, missing clinician referral forms (8 occurrences) were assumed to follow the clinical diagnosis (i.e., for a pain diagnosis with a missing referral form, it was assumed that the individual was referred for pain management; if both a pain diagnosis and the referral form were missing, it was assumed that the individual was not referred for pain management).

All participants received a standard UNIP evaluation before entry into the study. The first 10 study participants were randomized by a web-based system to receive or not receive the validated pain screen, the Brief Pain Inventory–Short Form (BPISF), to determine whether the screen influenced reporting of pain in the subsequent noninvasive physical examination. All subsequent participants received the BPISF. The BPISF, a 9-item self-administered questionnaire developed by the World Health Organization, represents a biopsychosocial model that evaluates the severity of an individual’s pain (severity score) and the impact of this pain on daily functioning (interference score). The BPISF has been validated [22] in 29 languages [23] and in vulnerable populations [24]. The diagnostic ability of the BPISF was assessed by predetermined standardized cutoff scores for both severity and interference. Scores of 1–4 corresponded to mild pain, 5–6 to moderate pain, and 7–10 to severe pain [25].

All participants then received a noninvasive physical examination (see S1 Text) by a pain specialist physician, the reference standard. The primary outcome was the diagnostic ability of the UNIP (index test) versus the BPISF, as compared to the reference standard.

The following procedures were aimed at minimizing bias: (1) trained evaluators performing the initial assessment with the UNIP (index test) were blinded to the study procedures (i.e., they performed their standard evaluations without knowledge of the study); (2) the pain specialist physician (reference standard) was blinded to the outcome of the initial UNIP evaluation and the BPISF; and (3) data from the initial UNIP assessment and the BPISF were not gathered by the principal investigator (GK) until all other study procedures were completed, to maintain blinding.

### Statistical analysis

It was hypothesized that the sensitivity of the UNIP (index test) to detect pain relative to the reference standard would be approximately 15%; however, a conservative estimate of 50% was utilized for statistical planning. With this conservative estimate of 50% with 50 participants, assuming that the pain specialist diagnosed pain in 80% (*N =* 40), it was determined that 29% was the minimal effect size detectable at 80% power. Therefore, the PainT study was designed to enroll 50 participants in order to have 80% power to detect a 29% difference in the sensitivity of the UNIP (index test) and the validated pain screen, as compared to the reference standard. At mid-enrollment of 25 participants, preplanned interim analysis was conducted (see S2 Text for study protocol). Results are being presented prior to enrolling 50 participants given the highly significant nature of the findings and the impact that these data may have on the standard evaluation of millions of refugee torture survivors globally. The sensitivity, specificity, positive predictive value, and negative predictive value were calculated for the UNIP and the BPISF and are reported along with 95% confidence intervals. The positive and negative likelihood ratios are also reported. The difference in the sensitivity of the UNIP and the sensitivity of the BPISF as compared to the reference standard was analyzed by a test of 2 proportions. Two-sided *p-*values of less than 0.05 were considered to indicate statistical significance. Analyses were performed with R software, version 3.5.3 (R Foundation, Vienna, Austria).

## Results

### Participants

**Fig 1:**
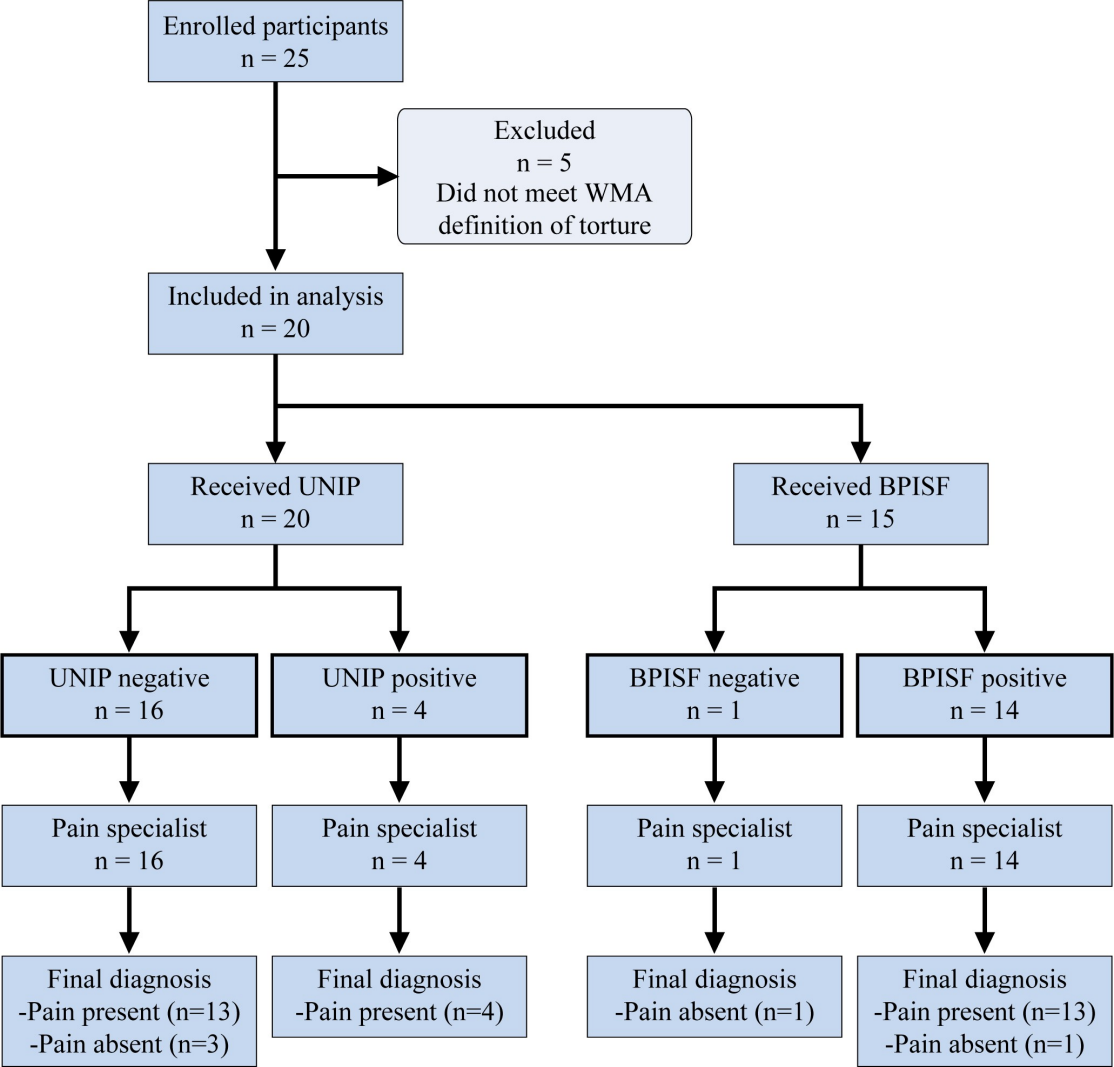
STARD enrollment flowchart. Twenty-five participants were enrolled. Five were excluded as they did not meet the WMA definition of torture, for a total of 20 unique participants who received the UNIP (index test); 15 of these participants received the BPISF. Pain specialist evaluation was the reference standard. BPISF, Brief Pain Inventory–Short Form; UNIP, United Nations Istanbul Protocol; WMA, World Medical Association.

Between February 1, 2017, and June 21, 2019, 25 participants were enrolled (Fig 1) and evaluated (Table 1). Three otherwise eligible individuals were excluded from enrollment due to language. In total, 90% of those contacted agreed to participate in the study, and enrollment was primarily limited by an inability to contact prospective participants (e.g., no email address, permanent address, or phone number). Interim analysis was conducted. Five participants were removed from analysis given that they did not meet the WMA torture definition on review of the UNIP assessment. A total of 20 participants (11 women and 9 men) were included in the analysis. Five participants received 2 UNIP evaluations (medical and/or psychiatric and/or gynecological) that were preplanned and based on trauma history, for a total of 25 unique evaluations included in the analysis. Licensed evaluators included internists, psychiatrists, psychologists, and gynecologists, and all had received training in the use of the UNIP. Participants were representative of a global population, with home countries in Africa, Central America, South Asia, the Caribbean, and the Middle East. Asylum applications were based on political persecution in 6 cases, gang violence in 5 cases, female genital mutilation/cutting (FGM/C) in 5 cases, persecution due to sexual orientation in 3 cases, and persecution due to race in 1 case. Methods of torture experienced were homogeneous, following the predictable pattern of systematic torture. No participants had unrelated baseline medical conditions that might have caused pain or confounded symptoms.

**Table 1:** PainT study participant data.

Enrollee number	Type of persecution	Type of torture	UNIP evaluation^Δ^	BPISF evaluation^□^	Pain specialist evaluation^◊^
1	Political	Shot, rifle assault	No^**∞**^	No	None
2	Gang	Beating, burned	No	N/A	Moderate–severe
3*	Political	Beating, assault	No^**∞**^/No^**∞**^	Yes	Moderate
5	Racial	Rape, assault	No	N/A	None
6*	Political	Beating, assault	No/No^**∞**^	Yes	Mild
7*	FGM/C, DV	FGM/C, beating, assault, rape, burned	Yes^**∞**^/No^†^	Yes	Moderate
8	LGBTQI	Beating, assault, near drowning	No^**∞**^	N/A	Severe
9*	FGM/C, DV	FGM/C, beating	No/Yes^**∞**^	N/A	Moderate
11	Gang	Beating, choking, rape	No	Yes	Severe
14	FGM/C, DV	Beating, assault, FGM/C, rape	No	N/A	Moderate–severe
15	Gang	Beating, choking	No	Yes	Severe
16	LGBTQI	Beating, assault	No	Yes	Mild
18	LGBTQI	Assault, knifing	No	Yes	None
19	Political	Beating, assault	Yes	Yes	Mild–moderate
20	FGM/C	FGM/C	No^†^	Yes	Severe
21*	Gang	Shooting	No^**∞**^/Yes	Yes	Moderate–severe
22	Political	Sexual assault, rape, beating	No	Yes	Moderate
23	FGM/C, DV	FGM/C, assault, knifing, rape	No^†^	Yes	Mild–moderate
24	Gang	Rape, beating	No	Yes	Severe
25	Political	Beating, assault, waterboarding	No	Yes	Mild–moderate

^Δ^Whether the UNIP evaluator diagnosed chronic pain and referred the participant for further evaluation or treatment.

^□^Whether the BPISF identified pain.

^◊^Severity of pain identified by the pain specialist.

*Participants had 2 preplanned UNIP evaluations based on trauma: 2 entries shown for UNIP column.

^†^Pain noted on the evaluation without the clinician making a diagnosis and referring the participant for further assessment or treatment.

^∞^Referral form missing, referral assumed to follow clinical diagnosis.

BPISF, Brief Pain Inventory–Short Form; DV, domestic violence; FGM/C, female genital mutilation/cutting; LGBTQI, lesbian, gay, bisexual, transgender, questioning, intersex; N/A, not applicable; UNIP, United Nations Istanbul Protocol.

All included participants were survivors of physical torture as defined by the WMA Declaration of Tokyo [20] and underwent standard medical evaluations guided by the UNIP. Perpetrators of violence included government officials, police and military personnel, and highly organized gangs.

### Test results

By the reference standard, 85% of participants (*N =* 17/20) were diagnosed with chronic pain.

**Fig 2:**
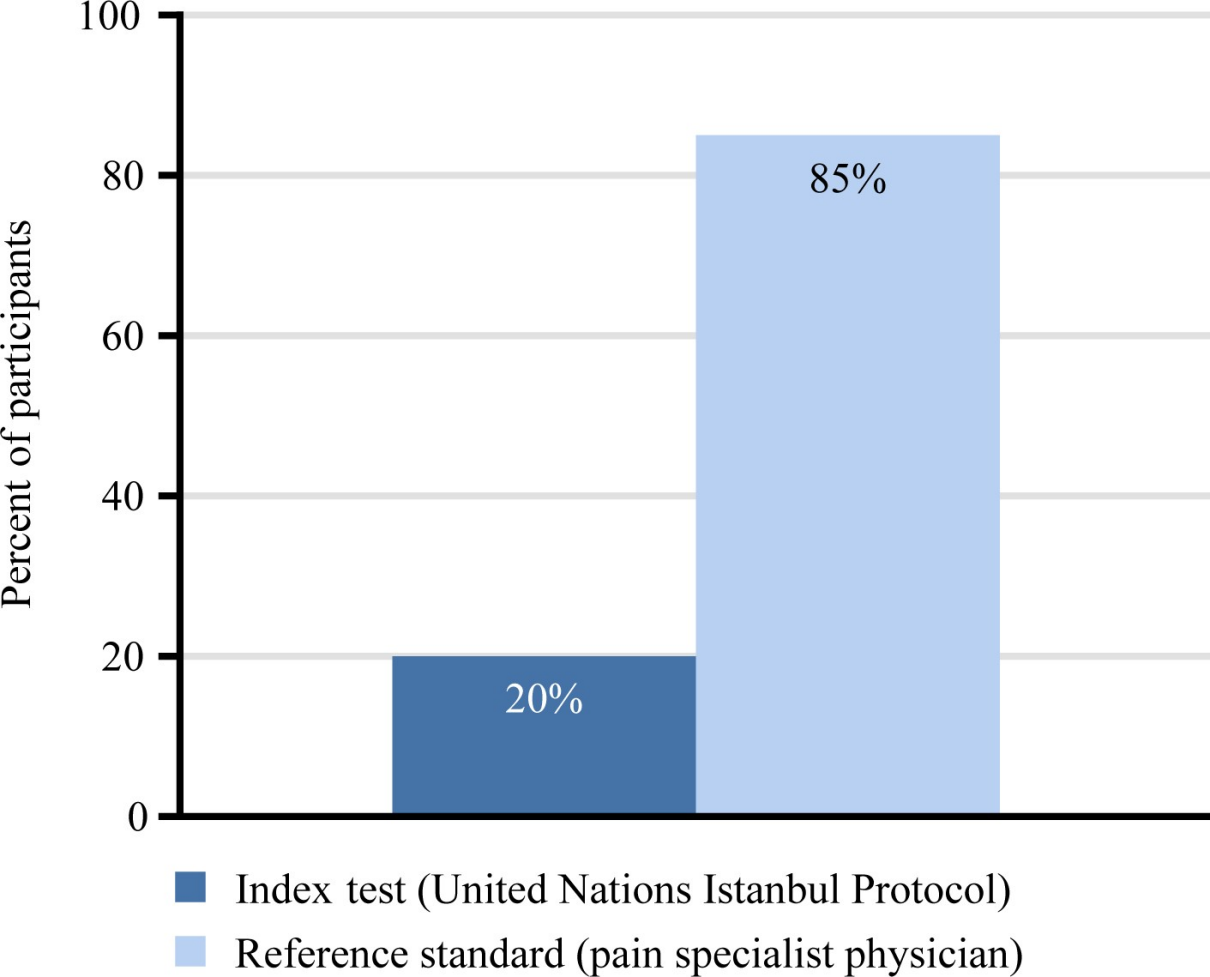
Evaluator’s ability to capture pain using the index test versus the reference standard.

By standard UNIP assessment, 28% (*N =* 7/25 [20 participants, 5 received 2 independent evaluations]) of evaluations noted chronic pain, and 8% (*N =* 2/25) of evaluations resulted in both a diagnosis of chronic pain and a referral for further evaluation or treatment. When grouped by type of persecution, the rate of identification of chronic pain for evaluators assessing FGM/C was 71% (*N =* 5/7 evaluations) as compared to the rate of identification by evaluators assessing persecution for political belief or sexual orientation or persecution by gang violence (11%, *N =* 2/18 evaluations). Of the individuals who experienced FGM/C and were noted to have chronic pain, 28% (*N =* 2/7) of evaluations resulted in a clinical diagnosis and referral for treatment. For the 5 participants who received 2 preplanned, independent evaluations based on trauma history (i.e., medical and/or psychiatric and/or gynecological), if either evaluation resulted in a diagnosis of chronic pain and referral, it was assumed that pain was captured, to conservatively maximize the diagnostic ability of the UNIP. Of the total 25 evaluations, 8 were missing a referral form. Accounting for missing forms, which were assumed to follow clinical diagnosis, a maximum of 16% (*N =* 4/25) of UNIP evaluations (or 20% of unique participants [*N =* 4/20]) received a diagnosis of chronic pain and a referral for further evaluation or treatment (Fig 2). Of the unique pain diagnoses (*N =* 17), 2 were of mild pain, 4 were of moderate pain, 5 were of severe pain, and 6 were of pain crossing more than 1 category, as determined by the reference standard. In total, 93% (*N =* 14/15) of participants who received the BPISF were diagnosed with chronic pain. Of these 14 participants, 9, 2, and 3 individuals were diagnosed with mild, moderate, and severe pain, respectively; 9, 2, and 2 individuals had mild, moderate, and severe interference of pain in their lives, respectively, with 1 individual having no appreciable pain interference. In the first 10 participants, completion of the BPISF did not increase reporting of pain in the subsequent noninvasive physical examination by a pain specialist, nor did qualitative information from the investigators indicate symptom exaggeration.

**Fig 3:**
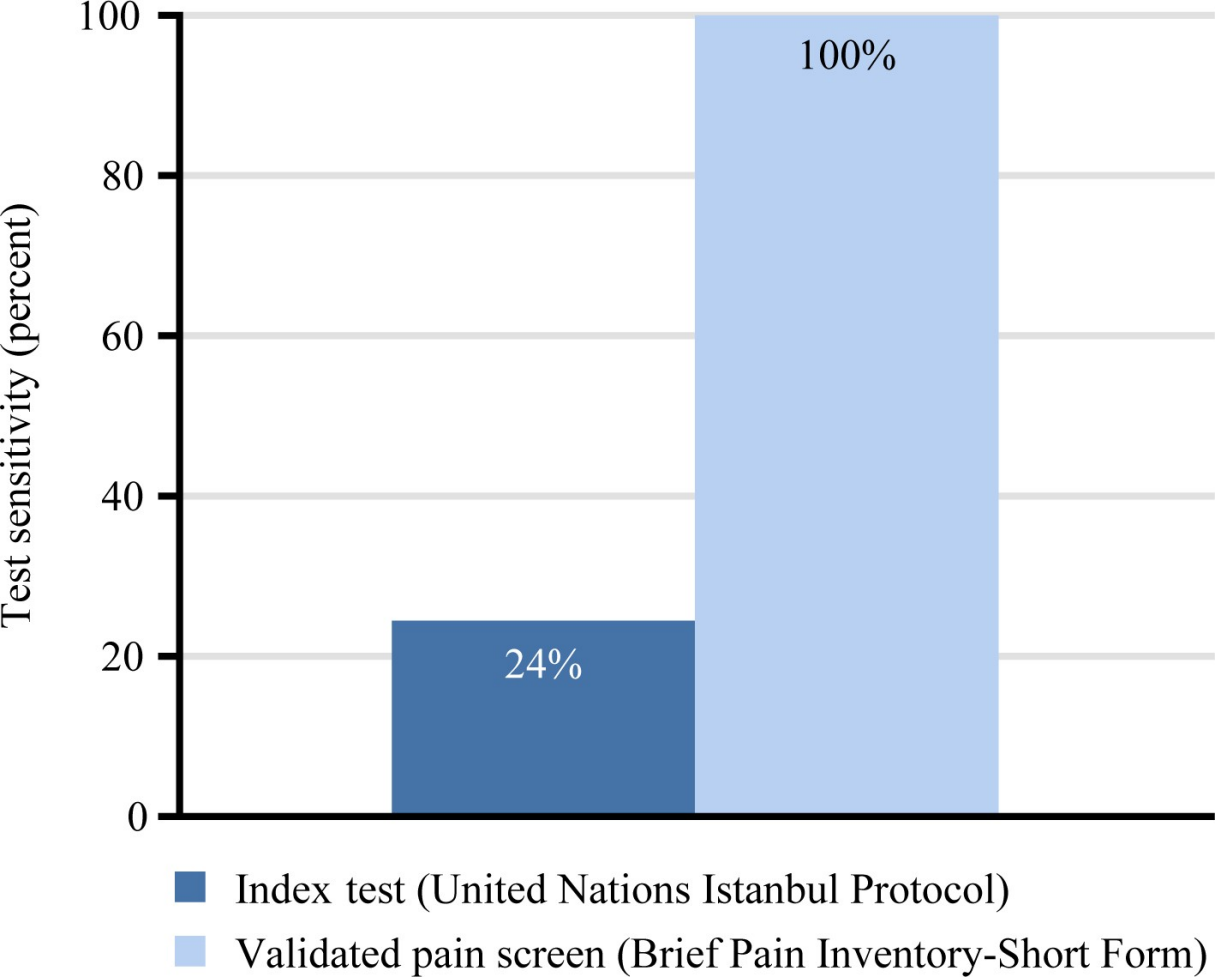
Sensitivity of index test versus the Brief Pain Inventory–Short Form (validated pain screen).

Of the 17 unique participants diagnosed with chronic pain according to the reference standard, 4 had pain captured by the UNIP, resulting in a sensitivity of 24% (95% CI 8%–50%), a specificity of 100% (95% CI 31%–100%), a negative predictive value of 19% (95% CI 5%–46%), and a positive predictive value of 100% (95% CI 40%–100%). Due to a specificity of 100%, the positive likelihood ratio was not calculated; the negative likelihood ratio was 0.76 (95% CI 0.59–1.00). Of the participants who received the BPISF and who were diagnosed with chronic pain according to the reference standard (*N =* 13), all were diagnosed with chronic pain according to the BPISF, resulting in a sensitivity of 100% (95% CI 72%–100%); the specificity was 50% (95% CI 9%–91%), negative predictive value was 100% (95% CI 5%–100%), and positive predictive value was 93% (95% CI 64%–100%). The positive likelihood ratio was 2.00 (95% CI 0.50–8.00), and the negative likelihood ratio was 0 (95% CI lower limit 0; upper limit not estimable). The difference in the sensitivity of the UNIP as compared to the BPISF was significant, with *p* < 0.001 (Fig 3).

No participants reported any adverse events from participating in the index test, the validated pain screen, or the reference standard examination.

## Discussion

We found that providers using only the standard UNIP (index test) captured pain in a maximum of 16% of evaluations, as compared to 85% of participants being diagnosed with chronic pain by the reference standard. When employed, the validated BPISF screen had a sensitivity of 100% and a negative predictive value of 100%, as compared to a sensitivity of 24% and negative predictive value of 19% for the UNIP. These data provide an impetus and mechanism to advance the standard of care of torture survivors by supplementing the standard global assessment protocol, the UNIP, with a screen for chronic pain. The authors recommend that all torture survivors who receive an evaluation by the UNIP also receive a screen for chronic pain.

Several limited studies in discrete populations of torture survivors demonstrate the high prevalence of pain, such as headache in up to 93% of survivors, musculoskeletal pain in up to 87% of survivors, and extremity pain in up to 72% of survivors [9]. This pain often correlates with the mechanism of injury: falanga leads to peripheral neuropathy, suspension from upper extremities results in brachial plexus injury, head trauma leads to trigeminal neuralgia, positional torture causes intervertebral disc herniation, and sexual assault and rape are correlated with abdominal, pelvic, genital, or anal pain [26]. Further described is complex regional pain syndrome after suspension from extremities, muscle crush injuries and severe disability after ghotna (roller crushing of muscles), and vertigo, loss of consciousness, and dizziness [11], amongst other symptoms resulting from torture. With an overall prevalence of persistent pain of approximately 87% among torture survivors, potentially 27 million people globally experience chronic pain resulting from torture [9].

Despite the documented high prevalence of chronic pain, our study’s findings suggest that evaluators using the UNIP fail to capture pain in a majority of torture survivors. Physical and psychological sequelae of torture occur in the context of complex trauma, superimposed on chronic medical conditions, making discrete medical diagnoses challenging [27]. Torture survivors flee their home countries due to persecution, often experiencing a loss of social status, financial difficulty, and migration trauma [28]. On arrival to a host country, they may be separated from their children, forced into detention, experience homelessness, lack access to medical care, face reactive xenophobia [29], and experience social isolation due to loss of community, language, and career [30,31], which have been demonstrated to lead to worse health outcomes [32]. Further limiting the diagnosis of pain, torture often aims to inflict the greatest trauma with minimal residual evidence [33], and the presence of physical findings suggesting an etiology of pain symptoms is low [34]. Additionally, the pathogenesis of pain after torture is not well understood. Dysfunctional spinal and supraspinal pain modulation, with increased excitability that persists after torture, is described [35], though studies are limited. Due to the absence of evidence-based diagnostic tools, the presence of concurrent PTSD or MD [36], a lack of physical findings, and a gap in the understanding of the pathophysiology of disease, pain in torture survivors is most often misdiagnosed and treated as a manifestation of psychological trauma. While somatization may be an alternative and adaptive mechanism to express psychological distress, particularly in individuals from cultures where mental illness is stigmatized [37], data from this study do not support the clinical assumption that pain after torture is exclusively a manifestation of psychological distress or that somatization should be a default diagnosis in this population.

In this study, 85% (*N =* 17/20) of individuals were diagnosed with chronic pain by the reference standard. Providers utilizing only the UNIP captured pain in a maximum of 16% (*N =* 4/25) of evaluations. In assuming that missing referral forms followed the clinical diagnosis and that, in repeat evaluations, if either evaluator diagnosed and referred for pain, it was captured, we maximized the presumed ability of providers utilizing the UNIP to capture pain. The true ability is likely less than 10%, given the raw rate of diagnosis and referral of 8% that we found. While evaluators assessing FGM/C were more accurate at identifying pain (71%, *N =* 5/7), there was still a lack of referral for further evaluation or treatment. Of the 18 total UNIP evaluations that missed positive pain diagnoses, 15 participants had moderate or severe pain, by the reference standard. This indicates that even debilitating pain is missed by providers who use only the UNIP. The type of pain varied, and included neuropathic, visceral, and somatic musculoskeletal pain. Sensitivity was selected as the most clinically relevant indicator, as the aim is to identify all torture survivors who experience chronic pain: In essence, false positives are more tolerable than false negatives, i.e., it is more detrimental to miss pain in torture survivors than for individuals without pain to screen positive and be referred for further evaluation. The significant sensitivity and negative predictive value differences between the UNIP (maximized at 24% and 19%, respectively) and the BPISF (100% and 100%, respectively) have clinical implications. These data suggest that integrating a validated pain screen into the UNIP may facilitate pain diagnoses by general evaluators.

Accurate diagnosis of pain is a critical component of treatment and rehabilitation. The physical and psychological sequelae of torture independently modulate each other, with physical trauma worsening psychological trauma and vice versa in a cycle of mutual maintenance [38]. This suggests that without addressing both physical and psychological trauma in torture survivors, rehabilitation will be limited. While rigorous studies in refugee torture survivors have not been conducted, trauma in this population mirrors that in other populations such as prisoners of war, concentration camp survivors, and war veterans [39]. Studies in these populations demonstrate that while trauma cannot be eliminated, rehabilitation after comorbid psychological and physical trauma is possible with a multidisciplinary approach [14].

Some limitations of the study merit consideration. The WMA definition of torture was used for the study, rather than the more restrictive United Nations Convention against Torture definition. However, the WMA definition is reported to be the most relevant to the medical profession, and studies have demonstrated that the applied difference is likely negligible [40]. Small sample size, the single-site nature of the study, and the exclusion of individuals who did not speak 1 of the 5 study languages are also limitations to the study. However, the WCCHR has evaluated individuals from 74 countries, and the 5 study languages—English, French, Spanish, Arabic, and Punjabi—represent the primary language spoken by 90% of the clinic’s population. As such, recruitment is likely representative of the global population of torture survivors. Further studies are warranted, particularly in the realm of pain management in tortured children, such as child soldiers.

In conclusion, our study found that the global standard assessment of torture survivors, the UNIP, can be supplemented by the use of a validated pain screen to increase the accuracy of chronic pain diagnosis.

## Supporting information

S1 STARD Checklist STARD checklist. (DOCX)

S1 Table Ten most common methods of torture. (DOCX)

S1 Text Noninvasive pain examination case report form. (PDF)

S2 Text Full study protocol. (PDF)

## Abbreviations

BPISF: Brief Pain Inventory–Short Form
FGM/C: female genital mutilation/cutting
MD: major depression
PTSD: post-traumatic stress disorder
UNIP: United Nations Istanbul Protocol
WCCHR: Weill Cornell Center for Human Rights
WMA: World Medical Association
