# Supplementary figures and images for: Chronic pain diagnosis in refugee torture survivors: A prospective, blinded diagnostic accuracy study
Source: PLoS Med. 2020 Jun 5;17(6):e1003108. doi: 10.1371/journal.pmed.1003108 (PMC7274371; doi:10.1371/journal.pmed.1003108)

**S1 Table.** Ten Methods may Account for up to 98% of Systematic Physical Torture


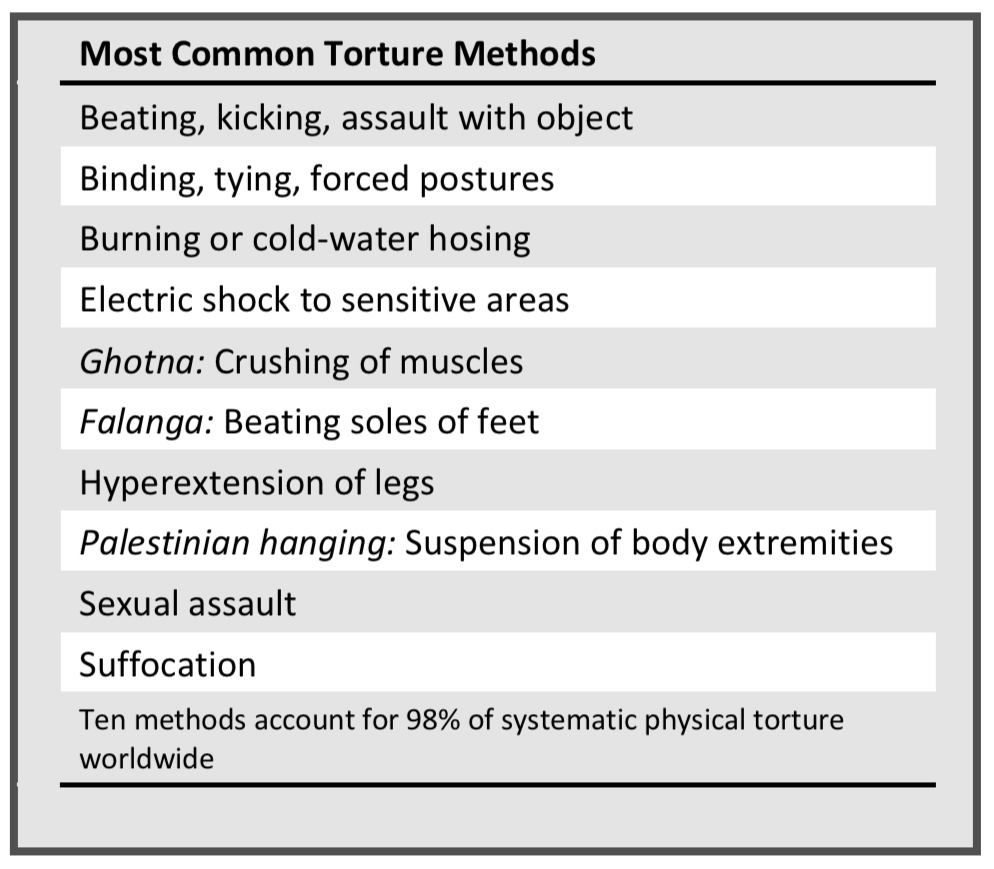

Supplement: S1 Table — (DOCX) [file pmed.1003108.s002.docx]
