# Supplementary material for: Chronic pain diagnosis in refugee torture survivors: A prospective, blinded diagnostic accuracy study
Source: PLoS Med. 2020 Jun 5;17(6):e1003108. doi: 10.1371/journal.pmed.1003108 (PMC7274371; doi:10.1371/journal.pmed.1003108)
Supplement: S1 Text — (PDF) [file pmed.1003108.s003.pdf]

Chronic pain diagnosis in refugee torture survivors: a prospective, blinded, diagnostic accuracy study

Subject Number	Subject Initials

Date Enrolled: ____________________

Site Location: ____________________

Completed By: ____________________

Chronic pain diagnosis in refugee torture survivors: a prospective, blinded, diagnostic accuracy study<br>PI: Gunisha Kaur, M.D. M.A.	Study ID: __ __ __<br>Subject Initials: __ __ __<br>Date: ___ / ___ / ______

CHIEF COMPLAINT:

____

____

____

____

____

____

____

DEMOGRAPHICS:

Age: ________ Years Old **Sex**: M F Other

Ethnicity:

☐ White ☐ Hispanic/Latino ☐ Black/African American ☐ Native American/American Indian

☐ Asian/Pacific Islander ☐ Other ___________________

Highest Education Achieved:

☐ Some High School ☐ High school graduate (or equivalent) ☐ Some college

☐ Trade/technical/vocational training ☐ College Graduate ☐ Post Graduate Degree

Professional or Employment Status

☐ Full Time ☐ Part Time ☐ Not Employed ☐ Retired ☐ Student

HISTORY OF PRESENT ILLNESS

Current Pain Intensity: ☐ None ☐ Mild ☐ Moderate ☐ Severe --- ☐ Increasing ☐ Decreasing

Pain Body Parts:

☐ Upper Back (L☐ /R☐)	☐ Lower Back (L☐ /R☐)	☐ Buttock (L☐ /R☐)	☐ Leg (L☐ /R☐)
☐ Neck (L☐ /R☐)	☐ Arm (L☐ /R☐)	☐ Shoulder (L☐ /R☐)	☐ Hip (L☐ /R☐)
☐ Knee (L☐ /R☐)	☐ Ankle (L☐ /R☐)	☐ Foot (L☐ /R☐)	☐ Abdomen (L☐ /R☐)
☐ Groin (L☐ /R☐)	☐ Flank (L☐ /R☐)	☐ Face (L☐ /R☐)	☐ Head (L☐ /R☐)
☐ Occipital Scalp (L☐ /R☐)	☐ Chest Wall (L☐ /R☐)		

Chronic pain diagnosis in refugee torture survivors: a prospective, blinded, diagnostic accuracy study<br>PI: Gunisha Kaur, M.D. M.A.	Study ID: __ __ __<br>Subject Initials: __ __ __<br>Date: ___ / ___ / ______

Duration of Pain: __________ ☐ Hours ☐ Days ☐ Weeks ☐ Months ☐ Years

Frequency of Pain:

☐ Constant ☐ Intermittent ☐ Intermittent with Exacerbation

☐ Worse in Morning ☐ Worse During Day ☐ Worse in Evening ☐ Worse at Night

☐ Worse on Weekends ☐ Worse in Winter ☐ No Pattern ☐ Worse after ________________________

Description/Quality of Pain:

☐ Constant ☐ Intermittent ☐ Burning ☐ Sharp

☐ Dull ☐ Stabbing ☐ Tender ☐ Tingling

☐ Aching ☐ Shooting ☐ Sore ☐ Throbbing

Additional Comments:

____

____

____

Pain Intensity with Activity (0 – 10): _______________

Pain Intensity at Rest (0 – 10): _______________

Highest Pain Intensity in the Last 24 Hours (0 – 10): _______________

Lowest Pain Intensity in the Last 24 Hours (0 – 10): _______________

Average Pain Intensity in the Last 24 Hours (0 – 10): _______________

Average Pain Intensity in the Last Week (0 – 10): _______________

What causes pain to increase?:

☐ Activity ☐ Cold ☐ Coughing ☐ Deep Breaths ☐ Defecation

☐ Eating ☐ Heat ☐ Laying Supine ☐ Sitting ☐ Sneezing

☐ Standing ☐ Urination ☐ Valsalva ☐ Walking

What causes pain to decrease?:

☐ Cold ☐ Heat ☐ Laying Supine ☐ Massage ☐ Sitting

☐ Standing ☐ Walking ☐ Medications ☐ Rest

How does the pain affect activities of daily living?:

☐ Difficult ☐ Requires Assistance ☐ Impossible ☐ Not a problem

Have you seen other doctors for this pain?: ☐ No ☐ Yes ________________________________

DIAGNOSES/TREATMENTS RELATED TO THIS PAIN:

Chronic pain diagnosis in refugee torture survivors: a prospective, blinded, diagnostic accuracy study<br>PI: Gunisha Kaur, M.D. M.A.	Study ID: __ __ __<br>Subject Initials: __ __ __<br>Date: ___ / ___ / ______

PAST MEDICAL HISTORY:

____

____

____

PAST SURGICAL HISTORY:

____

____

____

PAST FAMILY HISTORY:

____

____

____

PAST SOCIAL HISTORY:

____

____

____

MEDICATIONS:

____

____

____

ALLERGIES:

____

____

____

Chronic pain diagnosis in refugee torture survivors: a prospective, blinded, diagnostic accuracy study<br>PI: Gunisha Kaur, M.D. M.A.	Study ID: __ __ __<br>Subject Initials: __ __ __<br>Date: ___ / ___ / ______

REVIEW OF SYSTEMS

____

____

____

____

____

____

____

____

____

PHYSICAL EXAM

**HEAD:**<br>**NCAT:** ☐ Yes ☐ No<br>**Alopecia:** ☐ Yes ☐ No	**EMNT:**<br>**External Inspection of Ear and Nose:** ☐ Normal ☐ Abnormal<br>**Lips, Teeth, and Gums:** ☐ Normal ☐ Poor Dentition<br>**Oropharynx Exam:** ☐ Erythema<br>☐ Oral Pharynx Clear Without Erythema or Exudates
**EYES:**<br>**PERRL:** ☐ Yes ☐ No<br>**Extraocular Muscles Intact** ☐ Yes ☐ No<br>**Sclerae:** ☐ Normal ☐ Icterus	
**NECK:**<br>**General Neck Exam:**<br>☐ Normal ☐ Trachea Midline<br>☐ Full ROM ☐ No Lymphandenopathy<br>**Thyromegaly** ☐ Yes ☐ No<br>**Thyroid Palpable Nodule** ☐ Yes ☐ No	**RESPIRATORY:**<br>**Respiratory Effort:**<br>☐ Normal ☐ Using Accessory Muscles<br>☐ Labored Breathing<br>**Auscultation of Lungs:**<br>☐ Lungs Clear ☐ Crackles ☐ Rhonchi<br>☐ Wheezing ☐ ↓ Sound Breaths at R Base<br>☐ Friction Rub ☐ ↓ Sound Breaths at L Base

Chronic pain diagnosis in refugee torture survivors: a prospective, blinded, diagnostic accuracy study
PI: Gunisha Kaur, M.D. M.A.

Study ID: __ __ __
Subject Initials: __ __ __
Date: ___ / ___ / ______

CARDIOVASCULAR:

Auscultation of Heart:
☐ RRR No M/R/G ☐ Systolic Murmur
☐ Diastolic Murmur ☐ S3 ☐ S4

Pulses:
Radial: ☐ Intact ☐ Right ☐ Left
DP: ☐ Intact ☐ Right ☐ Left
PT: ☐ Intact ☐ Right ☐ Left

NEUROLOGIC:

Cranial Nerves: ☐ Normal ☐ II-XII Grossly Intact
Coordination: ☐ Normal ☐ No Asterixis
Spurling's: ☐ Positive ☐ Negative
☐ Left ☐ Right ☐ Bilaterally
SLR: ☐ Positive ☐ Negative
☐ Left ☐ Right ☐ Bilaterally

ABDOMEN/GI:

Inspection:
☐ Normal ☐ Proturbant ☐ Obese ☐ Surgical Scar(s)

Bowel Sounds:
☐ Normal ☐ Hyperactive ☐ Hypoactive ☐ Absent

Palpitation:
☐ Normal ☐ No Masses ☐ Nontender ☐ Nondistended ☐ Ascites
☐ Epigastric tenderness ☐ Suprapubic Tenderness ☐ Ventral Hernia ☐ Inguinal Hernia
☐ RUQ Tenderness ☐ LUQ Tenderness ☐ RLQ Tenderness ☐ LLQ Tenderness

Kidney:
☐ No CVA Tenderness ☐ R CVA Tenderness ☐ L CVA Tenderness

MUSCULOSKELETAL:

Gait: ☐ Normal ☐ Antalgic Gait
Tandem Gait: ☐ Normal ☐ Mildly Impaired ☐ Impaired
Heel Walking: ☐ Normal ☐ Abnormal
Toe Walking: ☐ Normal ☐ Abnormal

Spine:
☐ Normal Curvature ☐ Abnormal Scoliosis ☐ Abnormal Kyphosis ☐ Abnormal Lordosis

Cervical Flexion to: _______ **Degrees** ☐ With Pain ☐ Without Pain
Cervical Extension to: _______ **Degrees** ☐ With Pain ☐ Without Pain
Lumbar Flexion to: _______ **Degrees** ☐ With Pain ☐ Without Pain
Lumbar Extension to: _______ **Degrees** ☐ With Pain ☐ Without Pain

Cervical Paravertebral Muscular Tenderness: ☐ Absent ☐ Mild ☐ Moderate ☐ Severe
☐ Left ☐ Right ☐ Bilaterally
Lumbar Paravertebral Muscular Tenderness: ☐ Absent ☐ Mild ☐ Moderate ☐ Severe
☐ Left ☐ Right ☐ Bilaterally
Thoracic Paravertebral Muscular Tenderness: ☐ Absent ☐ Mild ☐ Moderate ☐ Severe
☐ Left ☐ Right ☐ Bilaterally

MUSCULOSKELETAL:

Facet Tenderness

Cervical: ☐ Absent ☐ Mild ☐ Moderate ☐ Severe
☐ Left ☐ Right ☐ Bilaterally

Thoracic: ☐ Absent ☐ Mild ☐ Moderate ☐ Severe
☐ Left ☐ Right ☐ Bilaterally

Lumbar: ☐ Absent ☐ Mild ☐ Moderate ☐ Severe
☐ Left ☐ Right ☐ Bilaterally

Sacroiliac: ☐ Absent ☐ Mild ☐ Moderate ☐ Severe

☐ Left ☐ Right ☐ Bilaterally

Greater Trochanteric Bursa: ☐ Present ☐ Not Present
☐ Left ☐ Right ☐ Bilaterally

Piriformis: ☐ Present ☐ Not Present
☐ Left ☐ Right ☐ Bilaterally

EXTREMITIES:

Digits: ☐ Normal ☐ No Clubbing ☐ No Cyanosis

Upper Extremities: ☐ Normal ☐ Abnormal

Lower Extremities: ☐ Normal ☐ Abnormal

Edema: ☐ +1 ☐ +2

Upper Extremity Power

C5 Elbow Flexion ☐ 1 ☐ 2 ☐ 3 ☐ 4 ☐ 5
☐ Left ☐ Right ☐ Bilaterally

C6 Wrist Extension ☐ 1 ☐ 2 ☐ 3 ☐ 4 ☐ 5
☐ Left ☐ Right ☐ Bilaterally

C7 Elbow Extension ☐ 1 ☐ 2 ☐ 3 ☐ 4 ☐ 5
☐ Left ☐ Right ☐ Bilaterally

C8 Finger Extension ☐ 1 ☐ 2 ☐ 3 ☐ 4 ☐ 5
☐ Left ☐ Right ☐ Bilaterally

T1 Finger Abduction ☐ 1 ☐ 2 ☐ 3 ☐ 4 ☐ 5
☐ Left ☐ Right ☐ Bilaterally

EXTREMITIES:

Lower Extremity Power

**L2 Hip Flexion**	☐ 1	☐ 2	☐ 3	☐ 4	☐ 5
	☐ Left		☐ Right		☐ Bilaterally
**L3 Knee Extension**	☐ 1	☐ 2	☐ 3	☐ 4	☐ 5
	☐ Left		☐ Right		☐ Bilaterally
**L4 Foot Dorsiflexion**	☐ 1	☐ 2	☐ 3	☐ 4	☐ 5
	☐ Left		☐ Right		☐ Bilaterally
**L5 Hallux Dorsiflexion**	☐ 1	☐ 2	☐ 3	☐ 4	☐ 5
	☐ Left		☐ Right		☐ Bilaterally
**S1 Hallux Plantar Flexion**	☐ 1	☐ 2	☐ 3	☐ 4	☐ 5
	☐ Left		☐ Right		☐ Bilaterally

SENSORY EXAM:

Sensation to…

Light touch:	☐ Normal	☐ Abnormal
Temperature:	☐ Normal	☐ Abnormal
Pinprick:	☐ Normal	☐ Abnormal

SKIN:

Sensation to…

Normal Skin Color:	☐ Yes	☐ No
Normal Skin Texture:	☐ Yes	☐ No
Rashes:	☐ Yes	☐ No
Lesions:	☐ Yes	☐ No
Normal Skin Turgor:	☐ Yes	☐ No

REFLEXES:

**Patella:**	☐ Absent	☐ 1+	☐ 2+	☐ 3+	☐ 4+
		☐ Left	☐ Right	☐ Bilaterally	
**Achilles:**	☐ Absent	☐ 1+	☐ 2+	☐ 3+	☐ 4+
		☐ Left	☐ Right	☐ Bilaterally	
**Brachioradialis:**	☐ Absent	☐ 1+	☐ 2+	☐ 3+	☐ 4+
		☐ Left	☐ Right	☐ Bilaterally	
**Bicep:**	☐ Absent	☐ 1+	☐ 2+	☐ 3+	☐ 4+
		☐ Left	☐ Right	☐ Bilaterally	
**Tricep:**	☐ Absent	☐ 1+	☐ 2+	☐ 3+	☐ 4+
		☐ Left	☐ Right	☐ Bilaterally	

Chronic pain diagnosis in refugee torture survivors: a prospective, blinded, diagnostic accuracy study<br>PI: Gunisha Kaur, M.D. M.A.	Study ID: __ __ __<br>Subject Initials: __ __ __<br>Date: ___ / ___ / ______

MENTAL STATUS:

**Orientation:**	☐ Alert	☐ Oriented x3		
**Affect:**	☐ Normal	☐ Blunted	☐ Flat	
	☐ Labile	☐ Anxious	☐ Fearful	☐ Tearful
**Mood:**	☐ Euthymic	☐ Sad	☐ Depressed	
	☐ Manic	☐ Euphoric	☐ Angry	
**Memory:**	☐ Normal	☐ Abnormal		

JOINT PAIN EXAM:

____

____

____

OTHER TESTS:

____

____

____

Chronic pain diagnosis in refugee torture survivors: a prospective, blinded, diagnostic accuracy study<br>PI: Gunisha Kaur, M.D. M.A.	Study ID: __ __ __<br>Subject Initials: __ __ __<br>Date: ___ / ___ / ______

FINAL ASSESSMENT

Assessment:

____

____

Diagnosis:

____

____

Chronic Pain ☐ Yes ☐ No

PAIN:

ICD 10 Code: ☐ None ____________________ **Pain Location(s):** ☐ None ____________________ **Type of Pain:** ☐ None ____________________

Mechanism of Injury: ☐ N/A

____

____

Pain consistent with mechanism of Injury: ☐ Yes ☐ No ☐ N/A

Secondary Injury: ☐ No ☐ Yes (please elaborate):

____

____

Medical Treatment for Pain: ☐ N/A

____

____

Physical Deformities: ☐ No ☐ Yes (please elaborate):

____

____

Confounding Medical Conditions: ☐ No ☐ Yes (please elaborate):

____
