# Supplementary material for: Chronic pain diagnosis in refugee torture survivors: A prospective, blinded diagnostic accuracy study
Source: PLoS Med. 2020 Jun 5;17(6):e1003108. doi: 10.1371/journal.pmed.1003108 (PMC7274371; doi:10.1371/journal.pmed.1003108)
Supplement: S2 Text — (PDF) [file pmed.1003108.s004.pdf]

Supplementary Material

Chronic pain diagnosis in refugee torture survivors: a prospective, blinded, diagnostic accuracy study

This supplement contains the following items:

1. Cover Page……………………………………………………………………….1
2. Original protocol…………………………………………………………………2
3. Final protocol……………………………………………………………………27
4. Summary of changes to protocol……………………………………………..46
5. Original statistical analysis plan……………………………………………...47
6. Final statistical analysis plan…………………………………………………49
7. Summary of changes to statistical analysis…………………………………..50

Gunisha Kaur M.D. M.A.
Assistant Professor of Anesthesiology
Co-Medical Director, Weill Cornell Center for Human Rights
Director, Anesthesiology Global Health Initiative
Program Director, Anesthesiology Global Health Fellowship

Weill Cornell Medicine
Department of Anesthesiology
525 East 68th Street, Box 124
New York, NY 10065

Final Protocol

Weill Cornell Medicine NewYork-Presbyterian

TITLE: The implementation of a novel pain-screening tool in the diagnoses of pain symptoms and syndromes in refugee torture survivors.

IRB Protocol #: 1608017472
Version Date: 6/18/2019
Funding Source(s): Weill Cornell Medicine Clinical and Translational Science Center KL2 Award

Principal Investigator:
Name: Gunisha Kaur, M.D., M.A.
Address: 525 East 68th Street, Box 124 New York, NY 10065
Telephone: 212-746-2461
E-mail address: gus2004@med.cornell.edu

Co-Investigators:
Name: Samantha Huynh, B.S.
Address: 525 East 68th Street, Box 124 New York, NY 10065
Telephone: 212-746-2952
E-mail address: sah4001@med.cornell.edu

Name: Michele Steinkamp, R.N.
Address: 525 East 68th Street, Box 124 New York, NY 10065
Telephone: 212-746-2953
E-mail address: mls9004@med.cornell.edu

Name: Alessandra Weidman
Address: 1300 York Avenue Room A-1014 New York, NY 10065
Telephone: 212-746-2243
E-mail address: mls9004@med.cornell.edu

Name: Roniel Weinberg
Address: 525 East 68th Street, Box 124 New York, NY 10065
Telephone: 212-746-2959
E-mail address: mls9004@med.cornell.edu

Statistician:
Name: Elizabeth Mauer, M.S.
Address: 425 East 61st Street New York, NY 10065
Telephone: 646-962-8026
E-mail address: elm2035@med.cornell.edu

Medical Monitor: N/A

Participating Sites: *Weill Cornell Medicine*

Table of Contents

CONFIDENTIALITY STATEMENT I
V

LIST OF ABBREVIATIONS I
V

1. PROTOCOL SUMMARY 1
1.1 Study Objectives 4
1.1.1 Objectives 4
1.1.2 Hypotheses / Research Questions 4

2. BACKGROUND AND SIGNIFICANCE 4

3. STUDY DESIGN AND METHODS 5
3.1 Overall Design 5
3.2 Interviews, Focus Groups, Surveys, and/or Observations 5

4. STUDY DESIGN 7
4.1 Study Population 7
4.2 Inclusion Criteria 7
4.3 Exclusion Criteria 7
4.4 Strategies for Recruitment and Retention 7

5. REGISTRATION PROCEDURES 8
5.1 Subject Registration (WCM only) 8

6. STUDY PROCEDURES 8
6.1 Schedule of Assessments 8

7.0 DATA REPORTING / REGULATORY CONSIDERATIONS 9
7.1 Data Collection 9
7.1.1 REDCap 9
7.2 Regulatory Considerations 9
7.2.1 Institutional Review Board/Ethics Committee Approval 9
7.2.2 Ethical Conduct of the Study 10
7.2.3 Informed Consent 10
7.2.4 Compliance with Trial Registration and Results Posting Requirements 10
7.2.5 Record Retention 11

8. STATISTICAL CONSIDERATIONS 1
1
8.1 Sample Size/Accrual Rate 11

8.2 Stratification Factors 11
8.3 Analysis of Endpoints 12
8.3.1 Analysis of Primary Endpoints 12
8.3.2 Analysis of Secondary Endpoints 12
8.4 Interim Analysis 12
8.5 Reporting and Exclusions 12

9. ADVERSE EVENT REPORTING REQUIREMENTS 1
2
9.1 Adverse Event Definition 12
9.1.1 Adverse Event Characteristics and Related Attributions
9.1.2 Recording of Adverse Events 12
9.1.3 Reporting of AE to WCM IRB 13
9.1.4 Reporting Events to Participants 13
9.1.5 Events of Special Interest 13
9.1.6 Reporting of Pregnancy 13

10. UNANTICIPATED PROBLEMS INVOLVING RISKS TO SUBJECTS OR *OTHERS* 1
3
10.1 Definition of Unanticipated Problems Involving Risks to Subjects or Others (UPIRTSO)
10.1.1 Unanticipated Problem Reporting

REFERENCES 1
4

Confidentiality Statement

This document is confidential and is to be distributed for review only to investigators, potential investigators, consultants, study staff, and applicable independent ethics committees or institutional review boards. The contents of this document shall not be disclosed to others without written authorization from WCM.

List of Abbreviations

**AE**	Adverse Event
**BPI-SF**	Brief Pain Inventory – Short Form
**CFR**	Code of Federal Regulations
**CRF**	Case Report Form
**CTSC**	Clinical Translational Science Center
**DSMB**	Data Safety Monitoring Board
**DSMP**	Data Safety Monitoring Plan
**FDA**	Food and Drug Administration
**GCP**	Good Clinical Practice
**HIPAA**	Health Insurance Portability and Accountability Act of 1996
**HRBFA**	Human Research Billing Analysis Form
**HUD**	Humanitarian Use Device
**ICF**	Informed Consent Form
**IDE**	Investigational Device Exemption
**IND**	Investigational New Drug
**IRB**	Institutional Review Board
**PHI**	Protected Health Information
**PI**	Principal Investigator
**REDCap**	Research Electronic Data Capture
**SAE**	Serious Adverse Event
**SUSAR**	Suspected Unexpected Serious Adverse Reaction
**UIRTSO**	Unanticipated Problem Involving Risks to Subjects or Others
**UNIP**	United Nations Istanbul Protocol
**WCCHR**	Weill Cornell Center for Human Rights
**WCM**	Weill Cornell Medicine
**WMA**	World Medical Association

1. Protocol Summary

We are evaluating refugee torture survivors who are receiving services at the Weill Cornell Center for Human Rights. There are two research questions in this study: if the current standard of care results in the under or missed diagnosis of pain and pain syndromes, and if a validated pain screening tool can supplement the current standard protocol used in the assessments of survivors of torture.

**Full Title:**	*The implementation of a novel pain-screening tool in the diagnoses of pain symptoms and syndromes in refugee torture survivors.*
**Short Title:**	*Pain after Torture*
**Principal Investigator:**	*Gunisha Kaur, M.D., M.A.*
**Study Description:**	*A prospective study investigating under or missed diagnoses of pain and pain syndromes that are identifiable by pain specialist physicians and/or a validated screening tool.*
**Sample Size:**	*N= 100*
**Enrollment:**	*This study will approximately screen 200 subjects and enroll 100 subjects.*
**Study Population:**	*Subjects seeking services at the Weill Cornell Center for Human Rights that have experienced torture from an authority figure, as per WMA definition.*
**Enrollment Period:**	*The subjects will be enrolled for about two hours.*
**Study Design:**	*In addition to the complex psychological sequelae resulting from torture, survivors of torture have pain and pain syndromes that are likely being under or undiagnosed in the current standard protocols for the evaluation of these individuals, that can be identified using a validated pain screening tool which triggers the referral to a pain specialist physician. There are two research questions being investigated in this study. The first is whether or not current standard protocols for the assessment of survivors of torture result in the under or missed diagnosis of pain and pain syndromes that are identifiable by pain specialist physicians. The second question is whether or not a validated pain screening tool can supplement the current standard protocols used in the assessments of survivors of torture, and can adequately indicate when patients should be referred to a pain physician for further evaluation. Standard procedures are limited to refugees undergoing a medical evaluation from a trained evaluator. Experimental and investigative procedures include using a validated self-assessment pain*

questionnaire (BPI-SF) to adequately indicate when patients should be referred to a pain physician for further evaluation.

The research plan consists of three major components:

1. *The subject indicates that they may be contacted by the research team about participating in a research study.*

2. *Researchers will call the potential subject and provide a brief introduction into the study. If the potential subject is interested in participating, an appointment will be scheduled at the CTSC. If the potential subject is only available during hours the CTSC is not open (before 8:00 AM and after 5:30 PM), a time will be scheduled to conduct the evaluation at 3 West. If necessary, a translator will be coordinated for the phone call.*

3. *At the 3 West or CTSC appointment, the informed consent process will be completed. The subject will complete the Brief Pain Inventory and will undergo a non-invasive pain assessment will be complete by a trained pain specialist physician. If necessary, a translator will be coordinated.*

Description of Sites/ Facilities Enrolling Participants:

The study will take place in the Clinical & Translational Science Center. If the subjects are only available when the CTSC is closed (before 8:00 AM or after 5:30 PM), their appointments will take place at 3 West.

Study Duration: *12/31/2022*

Participant Duration: *Participation will be limited to a one-time appointment to complete the study procedures, lasting approximately two hours.*

Primary Objective: *Evaluate whether or not current standard protocols for the assessment of survivors of torture result in the under or missed diagnosis of pain and pain syndromes that are identifiable by a pain specialist physician.*

Secondary Objectives: *Evaluate whether or not a validated pain screening tool can supplement the current standard protocols used in the assessments of survivors of torture, and can adequately indicate when patients should be referred to a pain physician for further evaluation.*

Exploratory Objectives: **N/A**

Endpoints: *The study endpoints will be the comparison of the UNIP and BPI-SF in accurately diagnosing pain as compared to the gold standard pain specialist evaluation.*

1.1 Study Objectives

The purpose of this study is to determine if the current standard of care results in the under or missed diagnosis of pain and pain syndromes, and if a validated pain screening tool can supplement the current standard protocol used in the assessments of survivors of torture.

1.1.1 Objectives

The Primary objective is to evaluate whether or not current standard protocols for the assessment of survivors of torture result in the under or missed diagnosis of pain and pain syndromes that are identifiable by a pain specialist physician.

The Secondary objective is to evaluate whether or not a validated pain screening tool can supplement the current standard protocols used in the assessments of survivors of torture, and can adequately indicate when patients should be referred to a pain physician for further evaluation.

1.1.2 Hypotheses / Research Questions

We hypothesize that the implementation of a validated pain screen, the Brief Pain Inventory – Short Form, can supplement the United Nations Istanbul Protocol to characterize the diagnosis of persistent pain in torture survivors as compared to the gold standard (a pain specialist evaluation).

2. Background and Significance

Torture leads to a combination of physical and psychological trauma. The literature on refugee survivors of torture demonstrates a high prevalence of chronic pain, with some data showing an incidence of 83%. As demonstrated, this pain may be persistent over a decade after trauma. A study by this investigator (WCM IRB # 1307014077) found the persistence of severe, debilitating chronic pain over two decades after torture in subjects from an immigrant South Asia population in NYC. A number of studies have detailed types of torture and their specific pain sequalae. For example, studies have demonstrated that falanga torture, or blunt trauma to the soles of the feet, may result in chronic pain, compensated gait, and peripheral neuropathy; that hanging from the limbs can be associated with brachial plexopathy, and that leg suspension or hyperextension can be correlated with lumbosacral plexus injury. A study of 133 asylum seekers found that physical symptoms were approximately twice as frequent as psychological symptoms, and were two to three times as frequent in survivors of torture as compared to non-tortured asylum seekers.

While psychiatric syndromes and somatization may contribute to chronic pain, several studies show that physical sequalae of torture accentuate psychological sequelae, rather than the other way around. Currently, the medical evaluation of survivors of torture is based on the "Istanbul Protocol: Manual on Effective Investigation and Documentation of Torture and Other Cruel, Inhumane or Degrading Treatment or Punishment," published by the United Nations. Though the Istanbul Protocol recommends a broad assessment of pain during the medical examination, this is not adequately being performed by physicians in their

evaluations of refugees. This may partly be due to the lack of use of a rapid screening tool for pain, similar to the screening tools used for psychiatric syndromes such as PTSD and MDD, which trigger evaluation by a specialist.

There is no current validated pain assessment being utilized by refugee clinics to prompt further questioning on pain or referral to a pain specialist physician. Without such a tool available to physicians who are not pain specialists, and with the existing overemphasis on the psychological components of refugee health, somatic pain diagnosis and treatment are predictably neglected. Further with the near complete lack of pain physician input, complicated conditions such as Complex Regional Pain Syndrome, typically go undiagnosed in this population. This prevents adequate treatment and rehabilitation of these patients. Given the rapidly increasing number of displaced individuals globally (reported as 68.5 million by the UN in 2017), a significant number of whom have suffered torture and experience chronic pain (5%-30% Burnett 2001), this project has the potential to impact thousands of individuals and establish a new standard of care. Beyond the refugee population, the screening tool has implications for anyone who has experienced a combination of physical and psychological trauma (for example, victims of sexual violence) who may not otherwise be referred to a pain specialist physician. This study is the foundation for a clinical trial to determine the outcome differences in survivors of torture when comparing standard treatment modalities to such modalities with the addition of somatic pain management. Treatment outcome differences would provide an impetus to integrate the pain screening tool into the standard evaluation of survivors of torture, and potentially into the Istanbul Protocol, to enhance clinical diagnoses and treatment. There are a limited number of regions in the US where such research can be conducted. Of the estimated 500,000 torture survivors in the US an approximated 75,000 to 90,000 reside in the New York City area. Additionally, Weill Cornell Center for Human Rights is one of the highest-volume clinics for refugees seeking asylum in US.

3. Study Design and Methods

3.1 Overall Design

This is a prospective, blind comparison to gold standard study, comparing the diagnosis of chronic pain in torture survivors using the standard United Nations Istanbul Protocol versus the novel application in this population of a validated pain screen.

3.2 Interviews, Focus Groups, Surveys, and/or Observations

A. *Administration*
   - *Timing and Frequency*
     The administration of the Brief Pain Inventory – Short Form (BPI) will take place following informed consent. The BPI should take a subject about 5-10 minutes to complete.
   - *Location*

The BPI will be administered in a CTSC or 3 West private space during their research appointment.

- *Procedures For Audio And Visual Recording*
  N/A
- *Person Identifiers*
  N/A

B. Study Instruments

- *We will be administering the Brief Pain Inventory-Short Form (BPI-SF). The BPI-SF is a validated self-assessment questionnaire and has been translated into the subjects' native language. Data will be described as N (%) or mean (sd). The UNIP and BPI-SF will be compared to the gold standard pain specialist evaluation by calculating sensitivity and specificity. 95% confidence intervals will be constructed for estimates of interest.*
- *The non-invasive pain evaluation will include an interview of the subject's history. It will be made clear in the beginning of the interview that anything the subject chooses to disclose will be confidential and will not affect their care or any services they have been offered through the Weill Cornell Center for Human Rights. The interviews will be recorded on a case report form without subject identifiers.*

4. Study Design

4.1 Study Population

Subjects seeking services at the Weill Cornell Center for Human Rights that have experienced torture from an authority figure.

4.2 Inclusion Criteria

Eligible subjects will be

1. *Above the age of 18*
2. *Speak English, French, Spanish, Arabic, or Punjabi as their primary language*
3. *Seeking services at the Weill Cornell Center for Human Rights*
4. *Have consented to being contacted by our research team*
5. *Have survived torture as defined by the WMA from authority figures*
    a. *"Torture" will be defined for this study as designated by the World Medical Association. "Authority" will be defined by government, police or military, or gang membership.*

4.3 Exclusion Criteria

1. *Asylum applicants through T visas (human trafficking), U visas (victims of abuse while in the US), VAMA (violence against women act), and SIJS (special immigrant juveniles), and pregnant women are excluded from the study.*
2. *Subjects who receive an evaluation that does not follow guidelines of the UN Istanbul Protocol will be excluded.*
3. *Subjects originally evaluated by the PI or other co-investigators of the study.*

4.4 Strategies for Recruitment and Retention

- <u>Anticipated accrual rate</u>
    - We anticipate to accrue 1-2 patients a month. Restrictions are due to the limited availability of potential subjects at WCCHR.

- We intend to enroll 100 subjects. All potential subjects will be contacted if they meet inclusion/exclusion criteria.

- <u>Source of participants</u>
    - Potential participants will be subjects seeking services at the Weill Cornell Center for Human Rights

- <u>Recruitment venues</u>
    - Recruitment for informed consent will be performed at the CTSC.

- How potential participants will be identified and approached
    - *The potential subject indicates that they may be contacted by the research team about participating in a research study.*
    - *Researchers will call the potential subject and provide a brief introduction into the study. If the potential subject is interested in participating, an appointment will be scheduled at the CTSC. If the potential subject is only available during hours the CTSC is not open (before 8:00 AM and after 5:30 PM), a time will be scheduled to conduct the evaluation at 3 West. If necessary, a translator will be coordinated for the phone call.*
    - *At the 3 West or CTSC appointment, the informed consent process will be completed. The subject will complete the Brief Pain Inventory and will undergo a non-invasive pain assessment will be complete by a trained pain specialist physician. If necessary, a translator will be coordinated.*

- Types of recruitment strategies planned
    - Phone consent
    - Informed consent in person
- Justification for Vulnerable Populations
    - The subject population are refugees, which are a vulnerable population. However, all safeguards will be in place to protect their identity. Data will be saved on a secure server and will be kept on a password-protected computer. Survey results and data will be identified by subject number and initials. Only personnel who are associated with the study will have access to the study specific records in the database. Additionally, mitigating factors include that subjects will already be connected to the Weill Cornell Center for Human Rights which provides medical and psychiatric support, as well as being connected to social workers and case managers who can help connect subjects to support systems.
- Subject Compensation
    - Subjects will be compensated with a $60 giftcard for their time at the completion of the study procedures.

5. Registration Procedures

5.1 Subject Registration (WCM only)

Subjects will *not* be registered within the WRG-CT as per the standard operating procedure for Subject Registration. This is because the subjects are not hospital patients and do not have a MRN to enroll onto OnCore.

6. Study Procedures

6.1 Schedule of Assessments

Table 1. Schedule of trial events

	Pre-Study	Visit 1
Phone consent	X	
Informed Consent		X
Demographics	X	
Medical history		X
Physical exam		X
BPI-SF		X

7.0 Data Reporting / Regulatory Considerations

7.1 Data Collection

The data collection plan for this study is to utilize REDCap to capture all treatment, toxicity, efficacy, and adverse event data for all enrolled subjects.

7.1.1 REDCap

REDCap (Research Electronic Data Capture) is a free data management software system that is fully supported by the Weill-Cornell Medical Center CTSC. It is a tool for the creation of customized, secure data management systems that include Web-based data-entry forms, reporting tools, and a full array of security features including user and group based privileges, authentication using institution LDAP system, with a full audit trail of data manipulation and export procedures. REDCap is maintained on CTSC-owned servers that are backed up nightly and support encrypted (SSL-based) connections. Nationally, the software is developed, enhanced and supported through a multi-institutional consortium led by the Vanderbilt University CTSA.

7.2 Regulatory Considerations

7.2.1 Institutional Review Board/Ethics Committee Approval

As required by local regulations, the Investigator will ensure all legal aspects are covered, and approval of the appropriate regulatory bodies obtained, before study initiation.

Before initiation of the study at each study center, the protocol, the ICF, other written material given to the patients, and any other relevant study documentation will be submitted to the appropriate Ethics Committee. Written approval of the study and all relevant study information must be obtained before the study center can be initiated or the IP is released to the Investigator. Any necessary extensions or renewals of IEC/IRB approval must be obtained for changes to the study, such as amendments to the protocol, the ICF, or other study documentation. The written approval of the IEC/IRB together with the approved ICF must be filed in the study files.

The Investigator will report promptly to the IEC/IRB any new information that may adversely affect the safety of the subjects or the conduct of the study. The Investigator will submit written summaries of the study status to the IEC/IRB as required. On completion of the study, the IEC/IRB will be notified that the study has ended.

All agreed protocol amendments will be clearly recorded on a protocol amendment form and will be signed and dated by the original protocol approving signatories. All protocol amendments will be submitted to the relevant institutional IEC/IRB for approval before implementation, as required by local regulations. The only exception will be when the amendment is necessary to eliminate an immediate hazard to the trial participants. In this case, the necessary action will be taken first, with the relevant protocol amendment following shortly thereafter.

Once protocol amendments or consent form modifications are implemented at the lead site, Weill Cornell Medicine, updated documents will be provided to participating sites. Weill Cornell Medicine must approve all consent form changes prior to local IRB submission.

Relevant study documentation will be submitted to the regulatory authorities of the participating countries, according to local/national requirements, for review and approval before the beginning of the study. On completion of the study, the regulatory authorities will be notified that the study has ended.

7.2.2 Ethical Conduct of the Study

The Investigators and all parties involved should conduct this study in adherence to the ethical principles based on the Declaration of Helsinki, GCP, ICH guidelines and the applicable national and local laws and regulatory requirements.

This study will be conducted under a protocol reviewed and approved by the applicable ethics committees and investigations will be undertaken by scientifically and medically qualified persons, where the benefits of the study are in proportion to the risks.

7.2.3 Informed Consent

The investigator or qualified designee must obtain documented consent according to ICH-GCP and local regulations, as applicable, from each potential subject or each subject's legally authorized representative prior to participating in the research study. Subjects who agree to participate will sign the approved informed consent form and will be provided a copy of the signed document.

The initial ICF, any subsequent revised written ICF and any written information provided to the subject must approved by IRB prior to use. The ICF will adhere to IRB/IEC requirements, applicable laws and regulations.

7.2.4 Compliance with Trial Registration and Results Posting Requirements

Under the terms of the Food and Drug Administration Modernization Act (FDAMA) and the Food and Drug Administration Amendments Act (FDAAA), the Sponsor-Investigator of the trial is solely responsible for determining whether the trial and its results are subject to the requirements for

submission to http://www.clinicaltrials.gov. Information posted will allow subjects to identify potentially appropriate trials for their disease conditions and pursue participation by calling a central contact number for further information on appropriate trial locations and trial site contact information.

7.2.5 Record Retention

Essential documents are those documents that individually and collectively permit evaluation of the study and quality of the data produced. After completion of the study, all documents and data relating to the study will be kept in an orderly manner by the Investigator in a secure study file. Essential documents should be retained for 2 years after the final marketing approval in an ICH region or for at least 2 years since the discontinuation of clinical development of the IP. In addition, all subject medical records and other source documentation will be kept for the maximum time permitted by the hospital, institution, or medical practice.

8. Statistical Considerations

8.1 Sample Size/Accrual Rate

We intend to evaluate the sensitivity and specificity of the existing qualitative, dichotomous screening diagnosis for pain, using the self-assessment pain questionnaire (BPI) and evaluation by pain physicians as gold standards. Ratings on the BPI greater than or equal to 6 will be classified as pain, as will a qualitative classification by a pain physician. We will first calculate the sensitivity and specificity of the current diagnostic exam, using the pain physician diagnosis as the gold standard. If the sensitivity is low, the current diagnostic tool will be ruled to be inadequate in assessing pain. We are focusing our analysis on the sensitivity rather than specificity of the test due to our belief that the current pain assessment is assigning an excess of false negative values.

We will then further evaluate the sensitivity and specificity of the existing diagnostic tool by using the BPI as the gold standard. A low sensitivity will rule that the current screening instrument is inadequate to diagnose pain. Finally, we will assess the sensitivity of the BPI by comparing it to the pain physician's evaluation, the latter of which will serve as the gold standard. This will be done to assess if the questionnaire is able to detect pain that would trigger a referral to a pain specialist. All analyses will be conducted in Stata 13 (StataCorp, College Station, TX).

Estimation of Sample Size. 100 subjects will be recruited. We hypothesize that 80% will be diagnosed with chronic pain by a pain specialist (gold-standard). To conservatively maximize the width of the obtained confidence interval, we assume that the sensitivity of the Istanbul Protocol is 50%; given this, we can construct a 95% confidence interval for the true sensitivity of the Istanbul Protocol to detect chronic pain between 39% to 61% (+/- 11%). However, based on our preliminary studies, the ability of the Istanbul Protocol to detect chronic pain is approximately 15%, which results in a more precise 95% confidence interval between 3% and 17% (+/- 7%). Based on our preliminary studies, we expect the sensitivity of the BPI to detect pain to be approximately 85%, with a 95% confidence interval between 78% and 92% (+/- 7%). All analyses will be conducted in R version 3.6.0 (Vienna, Austria).

8.2 Stratification Factors

N/A

8.3 Analysis of Endpoints

8.3.1 Analysis of Primary Endpoints

Data Analysis. Data will be described as N (%) or mean (sd). The UNIP and BPI-SF will be compared to the gold standard pain specialist evaluation by calculating sensitivity and specificity. 95% confidence intervals will be constructed for estimates of interest.

8.3.2 Analysis of Secondary Endpoints

N/A

8.4 Interim Analysis

Interim analysis will be performed to determine if the current study trajectory is on track.

8.5 Reporting and Exclusions

N/A

9. Adverse Event Reporting Requirements

Adverse events will not be reported following study procedures as the subjects are not patients, and would be lost to follow-up after the one time study appointment. This study is minimal risk, and we do not expect any adverse events as defined below. However, all unanticipated adverse events will be reported to the study PI within 24 hours and to the IRB as appropriate.

Adverse event (AE) monitoring and reporting is a routine part of clinical research. Safety is monitored by evaluation of adverse events reported by subjects or observed by investigators or research staff.

9.1 Adverse Event Definition

Any undesirable experience associated with a drug or procedure, also sometimes described as a side effect or negative reaction. Adverse events can range from mild to severe. Serious adverse events are those that can cause temporary or permanent disability and may result in hospitalization or death.

9.1.2 Recording of Adverse Events

All adverse events will be recorded on a subject specific AE log. The AE log will be maintained by the research staff and kept in the subject’s research chart.

9.1.3 Reporting of AE to WCM IRB

All AEs occurring on this study will be reported to the IRB according to the IRB policy, which can be accessed via the following link: http://researchintegrity.weill.cornell.edu/forms_and_policies/forms/Immediate_Reporting_Policy.pdf.

9.1.4 Reporting Events to Participants
N/A

9.1.5 Events of Special Interest

N/A

9.1.6 Reporting of Pregnancy

N/A

10. Unanticipated Problems Involving Risks to Subjects or *Others*
Not Applicable

References

1. Williams AC, Amris K. Pain from torture. Pain. 2007 Dec; 133(1-3):5-8. PMID: 18006232.
2. Thomsen AB, Eriksen J, Smidt-Nielsen K. Chronic pain in torture survivors. Forensic Sci Int. 2000 Feb 28; 108(3):155-63. PMID: 10737462.
3. Office of the United Nations High Commissioner for Human Rights. Istanbul Protocol: Manual on Effective Investigation and Documentation of Torture and Other Cruel, Inhumane or Degrading Treatment of Punishment. United Nations, New York, 2004.
4. Mollica RF, McInnes K, Sarajlic N, Lavelle J, Sarajlic I, Massagli MP. Disability associated with psychiatric comorbidity and health status in Bosnian refugees living in Croatia. JAMA. 1999 Aug 4; 282(5):433-9. PMID: 10442658.
5. Crosby SS. Primary care management of non-English speaking refugees who have experienced trauma: a clinical review. JAMA. 2013 Aug 7; 310(5):519-28. PMID: 23925622.

Supplementary Material

Chronic pain diagnosis in refugee torture survivors: a prospective, blinded, diagnostic accuracy study

Summary of Changes to Protocol:

There have been a number of amendments to the protocol between the initial approved protocol and final protocol to add and remove co-investigators and make minor changes to the methods. Most importantly, the format of our IRB has changed, therefore the formatting of the initial and final protocols differ.

- The initial protocol was only approved for English speaking subjects until the translated informed consent forms and survey tools were submitted and approved in a future amendment.
- We added compensation to adequately cover the subjects' time for participation and transportation costs.
- The Brief Pain Inventory randomization was removed.
- We also updated the protocol to recruit Arabic and Punjabi speaking subjects.

Gunisha Kaur M.D. M.A.
Assistant Professor of Anesthesiology
Co-Medical Director, Weill Cornell Center for Human Rights
Director, Anesthesiology Global Health Initiative
Program Director, Anesthesiology Global Health Fellowship

Weill Cornell Medicine
Department of Anesthesiology
525 East 68th Street, Box 124
New York, NY 10065

Initial Statistical Analysis

Chronic pain diagnosis in refugee torture survivors: a prospective, blinded, diagnostic accuracy study

We intend to evaluate the sensitivity and specificity of the existing qualitative, dichotomous screening diagnosis for pain, using the self-assessment pain questionnaire (BPI) and evaluation by pain physicians as gold standards. Ratings on the BPI greater than or equal to will be classified as pain, as will a qualitative classification by a pain physician. We will first calculate the sensitivity and specificity of the current diagnostic exam, using the pain physician diagnosis as the gold standard. If the sensitivity is low, the current diagnostic tool will be ruled to be inadequate in assessing pain. We are focusing our analysis on the sensitivity rather than specificity of the test due to our belief that the current pain assessment is assigning an excess of false negative values. We will then further evaluate the sensitivity and specificity of the existing diagnostic tool by using the BPI as the gold standard. A low sensitivity will rule that the current screening instrument is inadequate to diagnose pain. Finally, we will assess the sensitivity of the BPI by comparing it to the pain physician¿s evaluation, the latter of which will serve as the gold standard. This will be done to assess if the questionnaire is able to detect pain that would trigger a referral to a pain specialist. All analyses will be conducted in Stata 13 (StataCorp, College Station, TX).

We will target all patients seeking care at the Weill Cornell Center for Human Rights. The WCMC Center for Human Rights evaluates between 50-100 patients per year, making our initial population of study subjects at approximately N=100. We expect to gain cooperation from 75% of patients, resulting in an estimated total of N=75. Randomization will be into two groups: a treatment group administered the Brief Pain Inventory Short Form, a validated pain screening tool, as well as a non-invasive pain evaluation by a pain specialist physician, and a control group only receiving a non-invasive pain evaluation by a pain specialist physician. This will result in approximately N=37 per group. Assuming that the Brief Pain Inventory Short Form results in a 30% absolute increase in diagnoses of pain (versus administering solely the current protocol), we estimate that a sample of N=37 per group will be necessary. To test our primary objective of assessing the efficacy of an adjunctive pain evaluation in detecting pain or pain syndromes, we will administer the Brief Pain Inventory Short Form to the treatment group. Next, a pain specialist physician will conduct a physical exam to both the treatment and control groups for diagnosing pain and pain syndromes at the beginning of the patient's initial provider encounter.

To test our secondary objective, we will then evaluate the efficacy of the non-invasive pain evaluation (control group) and experimental instruments plus a non-invasive pain evaluation (treatment group) in determining which patients should be referred to a pain specialist physician for further evaluation. Descriptive statistics (frequencies, percentages, etc.) will be used to assess relevant patient demographics and components of clinical evaluations, including the questions on the Brief Pain Inventory Short Form. In order to analyze our primary outcome, we will conduct Chi-square or Fisher’s exact tests to test for significant differences between the experimental and control treatments in identifying pain. We will also use Chi-square or Fisher's exact tests to

analyze our secondary outcome: to detect differences between diagnoses of pain (based on the Brief Pain Inventory Short Form and/or standard procedures) and a diagnosis of pain or a pain syndrome by a pain specialist physician. All p-values will be one-sided, and significance will be evaluated at the 0.05 alpha level. All analyses will be conducted in Stata IC, Version 13 (College Station, TX, USA).

Final Statistical Analysis

Chronic pain diagnosis in refugee torture survivors: a prospective, blinded, diagnostic accuracy study

We intend to evaluate the sensitivity and specificity of the existing qualitative, dichotomous screening diagnosis for pain, using the self-assessment pain questionnaire (BPI) and evaluation by pain physicians as gold standards. Ratings on the BPI greater than or equal to 6 will be classified as pain, as will a qualitative classification by a pain physician. We will first calculate the sensitivity and specificity of the current diagnostic exam, using the pain physician diagnosis as the gold standard. If the sensitivity is low, the current diagnostic tool will be ruled to be inadequate in assessing pain. We are focusing our analysis on the sensitivity rather than specificity of the test due to our belief that the current pain assessment is assigning an excess of false negative values.

We will then further evaluate the sensitivity and specificity of the existing diagnostic tool by using the BPI as the gold standard. A low sensitivity will rule that the current screening instrument is inadequate to diagnose pain. Finally, we will assess the sensitivity of the BPI by comparing it to the pain physician's evaluation, the latter of which will serve as the gold standard. This will be done to assess if the questionnaire is able to detect pain that would trigger a referral to a pain specialist. All analyses will be conducted in Stata 13 (StataCorp, College Station, TX).

Estimation of Sample Size. 100 subjects will be recruited. We hypothesize that 80% will be diagnosed with chronic pain by a pain specialist (gold-standard). To conservatively maximize the width of the obtained confidence interval, we assume that the sensitivity of the Istanbul Protocol is 50%; given this, we can construct a 95% confidence interval for the true sensitivity of the Istanbul Protocol to detect chronic pain between 39% to 61% (+/- 11%). However, based on our preliminary studies, the ability of the Istanbul Protocol to detect chronic pain is approximately 15%, which results in a more precise 95% confidence interval between 3% and 17% (+/- 7%). Based on our preliminary studies, we expect the sensitivity of the BPI to detect pain to be approximately 85%, with a 95% confidence interval between 78% and 92% (+/- 7%). All analyses will be conducted in R version 3.6.0 (Vienna, Austria).

Supplementary Material

Chronic pain diagnosis in refugee torture survivors: a prospective, blinded, diagnostic accuracy study

Summary of Changes to Statistical Analysis:

With the removal of the randomization scheme, the statistical analysis plan was edited.

- The sensitivity and specificity of the BPI compared to the gold standard pain specialist physician analysis remained the same.
- Based on our updated power analysis, sample size was recalculated at 50 subjects; recruitment opened with enrollment to 100 subjects.

Gunisha Kaur M.D. M.A.
Assistant Professor of Anesthesiology
Co-Medical Director, Weill Cornell Center for Human Rights
Director, Anesthesiology Global Health Initiative
Program Director, Anesthesiology Global Health Fellowship

Weill Cornell Medicine
Department of Anesthesiology
525 East 68th Street, Box 124
New York, NY 10065
